# Persistent headaches sometimes concern incidental findings: A rare case of internal jugular vein agenesis in a 32‐year‐old man

**DOI:** 10.1002/ccr3.6423

**Published:** 2022-10-06

**Authors:** Mohammad Ashraful Amin, Sabrina Nahin, Mohammad Delwer Hossain Hawlader

**Affiliations:** ^1^ Department of Public Health North South University Dhaka Bangladesh; ^2^ Public Health Professional Development Society (PPDS) Dhaka Bangladesh; ^3^ Department of Physiology Green Life Medical College Hospital Dhaka Bangladesh

**Keywords:** agenesis, headache, internal jugular vein

## Abstract

Absence of an internal jugular vein at birth is infrequent. These developmental anomalies affect approximately 0.05 percent to 0.25 percent of the population in the general community. Avascular abnormalities emerging from the internal jugular vein were detected during radiographic studies of chronic headache in an adult male patient. A dull headache troubled a 32‐year‐old man for more than 20 years. After taking most of NSAIDs and other medications for the condition, which persisted, a diagnosis of left internal jugular vein agenesis was made, which was most likely the cause of the headaches. When treating recurrent, persistent headaches in the emergency room and outdoor medical services, keep in mind that agenesis of the jugular venous system can play a role—one of the uncommon causes of headaches we have observed in our cases.

## Graphical abstract

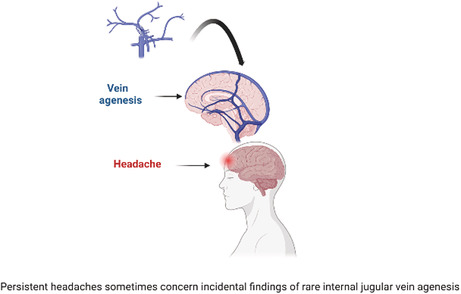

## 1 INTRODUCTION

The internal jugular vein (IJV) is a significant factor leading to venous drainage from intracranial components, draining blood from the head and neck region. The absence of an internal jugular vein (IJV) is an asymptomatic, relatively unusual vascular abnormality.^1^ In the general community, 0.05%–0.25% of people have developmental venous abnormalities.^2^ Most venous abnormalities are asymptomatic and usually found in the head and neck area.^3^ It is common for cerebral venous abnormalities to coexist with vascular anomalies in the head and neck region.^4^ Vascular tumors (hemangioma, hemangioendotelioma, and angiosarcoma) and vascular malformations (hemangioma, hemangioendotelioma, and angiosarcoma) are two types of vascular malformations (Table 1).^5^ Even though developmental venous anomalies (DVAs) are generally thought to be innocuous vascular malformations,^6^ We have met patients with symptoms of headache as DVAs might feature. Internal jugular vein absence is a unique congenital condition. Only a few cases (in the low double digits) of a missing internal jugular vein have been described in English literature to our awareness. Most cases were discovered by chance during a routine check before central venous cannulation in patients who received major surgery or radiologic.^2^ The vascular system's development defect known as arterio‐venous malformations (AVMs) is characterized by tangles of terribly developed blood vessels where the feeding arteries are joined straight to the venous drainage network without any intervening capillary system.^7^ The arteries transport the brain's oxygen‐rich blood from the heart. The oxygen‐depleted blood is returned to the heart and lungs through veins. This crucial procedure is hampered by a brain AVM. A brain AVM can trigger signs and symptoms like headaches or seizures in certain people.^8^ Vascular malformations are congenital abnormalities that have difficult‐to‐manage clinical manifestations. With contradicting information in the literature, their classification and treatment options have significantly changed over time.^9^

**TABLE 1:** ISSVA classification of vascular anomalies

Tumors	Malformations
Hemangioma Hemangioendothelioma Angiosarcoma Miscellaneous	Slow Flow
	Capillary Lymphatic Venous
	Fast‐flow
	Arterial Combined

One of the most common bodily complaints in people is a headache. Despite the fact that headaches are mostly benign, neuroimaging tests are regularly carried out in clinical settings because of concern that they may fail to detect a serious underlying condition. So, we report a 32‐year‐old man with left internal jugular vein agenesis who has experienced a dull headache for more than 20 years and who has no family history of the condition. There was not yet a remarkable intervention for this situation. We hypothesis this persistent headache may be an uncommon cause of internal vein agenesis.

## 2 CASE REPORT

**FIGURE 1:**
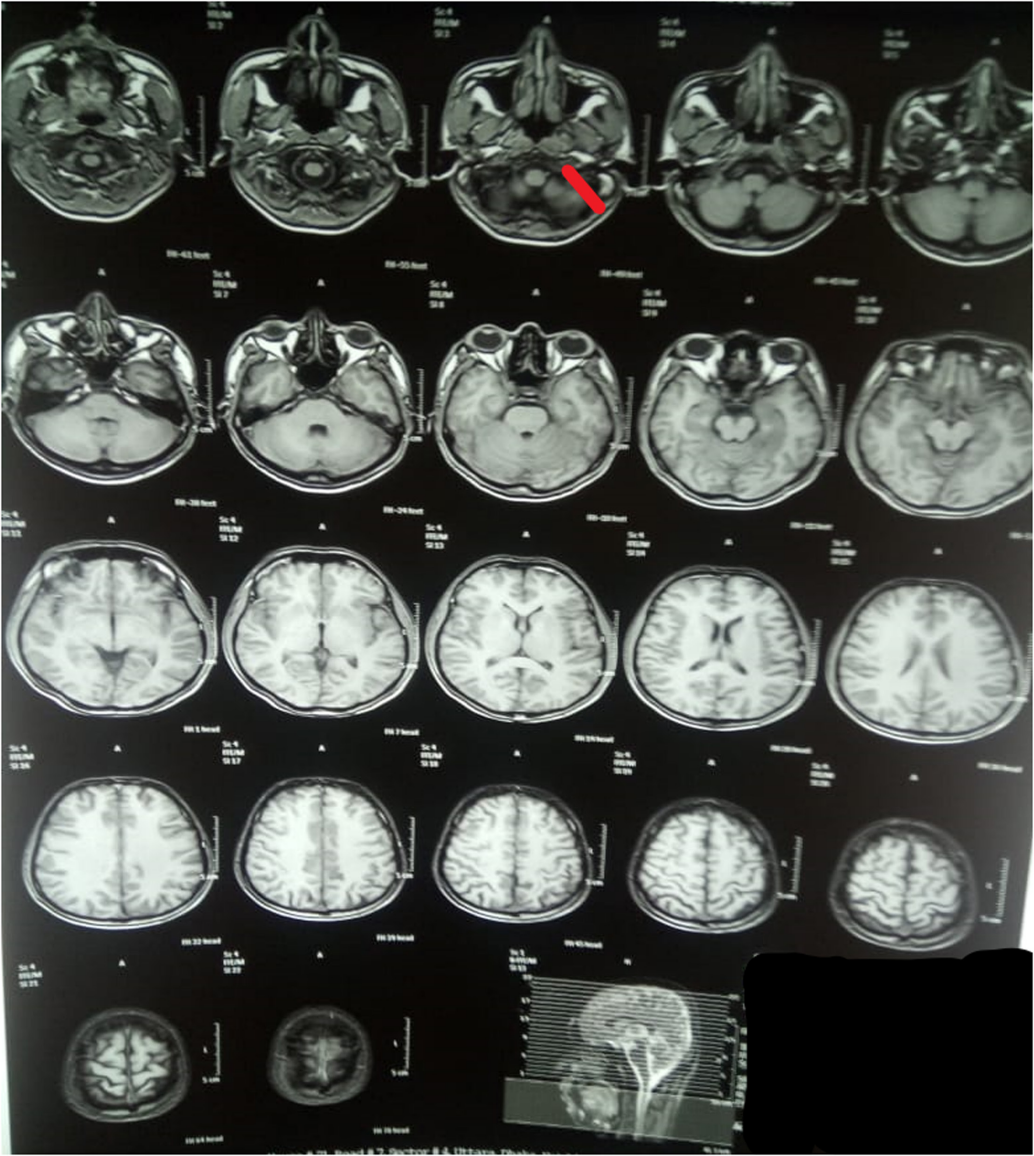
Magnetic resonance imaging (MRI) scan demonstrating absence of enhancement in the left internal jugular vein (IJV).

**FIGURE 2:**
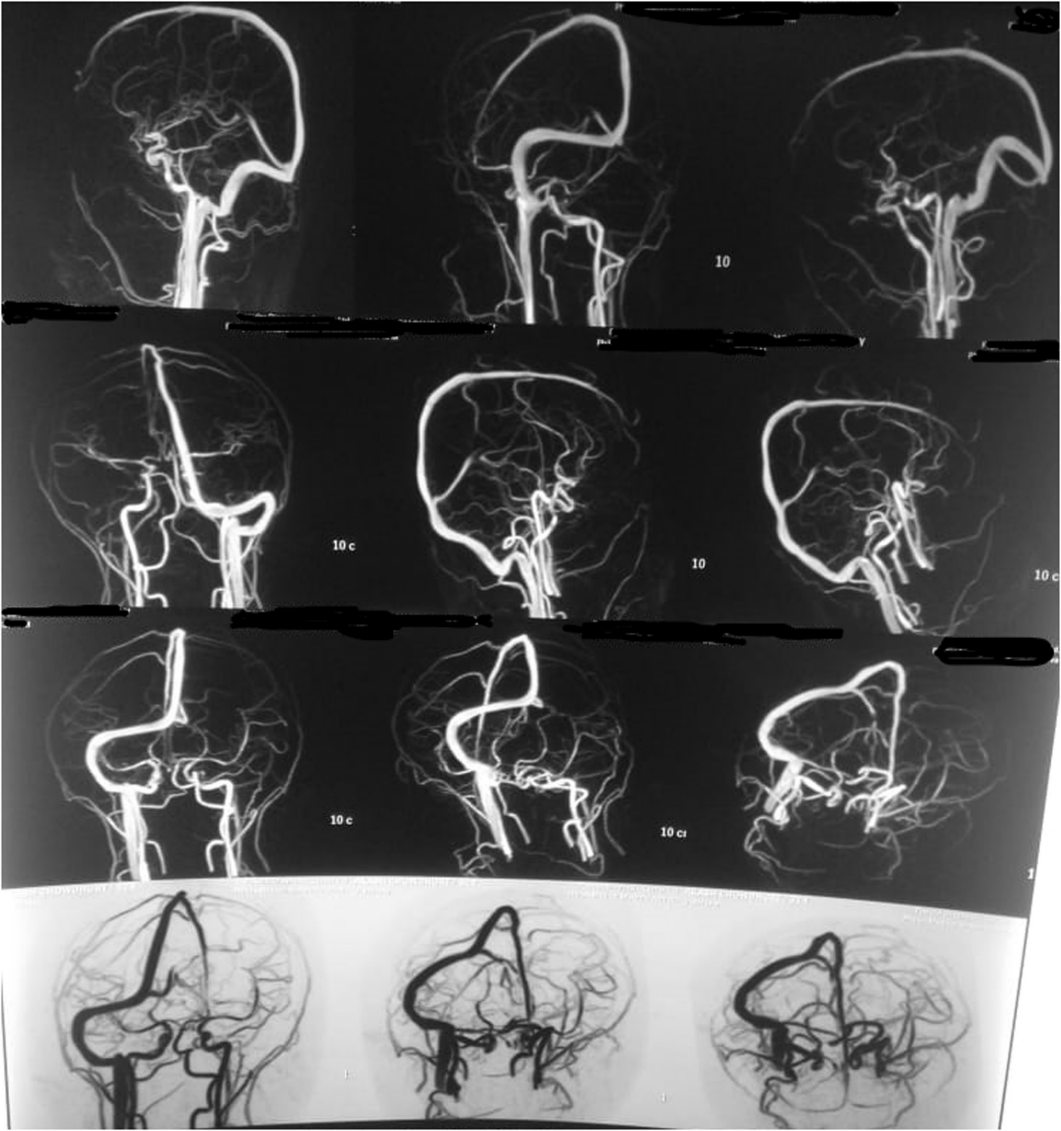
Magnetic Resonance Venography (MRV): Sagittal view of collateral system (dynamic magnetic resonance image) of the case: Revealed the jugular venous system of the left side is not visualized and the right side jugular venous system is prominent and deep veins and dural venous sinuses appear normal which suggestive of congenital agenesis of jugular venous system of left side.

A 32‐year‐old man with a 20‐year history of chronic headaches was referred to the National Institute of Neurosciences & Hospital on November 25th, 2021. Apart from that, the physical checkup was normal. Since 2002, he has had constant and recurring headaches, occurring 2–3 times each week. The headaches were reported as forehead pain that did not extend to his eye or any other body part. The attacks lasted 30 to 45 min on average. These headaches frequently occurred in the afternoon and were not characterized by eye redness or weeping. The headaches were so bad that he could not stand it any longer. Except for NSAIDs, which only eased the pain to a minor degree, no therapies were helpful before consulting with a physician. He was previously diagnosed with tension headaches and also treated as migraine for dull on–off headaches. He had previously been prescribed paracetamol, NSAIDs, propranolol, methysergide, antihistamines, anti‐anxiety and sedative medication and naproxen sodium were among the earlier ineffectual treatments. A clinical interview, the Minnesota Multiphasic Personality Inventory‐2 (MMPI‐2), the State–Trait Anxiety Inventory (STAI), and the Beck Depression Inventory (BDI) were all used in a comprehensive psychiatric assessment. The psychological perception was the situational anxiety resulting from his disease, with no underlying psycho‐emotional dysfunction. On numerous occasions, physicians have recommended that he undergo contrast‐enhanced computed tomography (CT scan) and magnetic resonance imaging (MRI), but he has always planned to do it later. He experienced acute watery diarrhea recently on November 10, 2021, and laboratory tests are done (Table 2). During this acute diarrhea period, he had associated with severe headache so he underwent contrast‐enhanced computed tomography (CT scan) (Figure 1) and Magnetic Resonance Venography (MRV) (Figure 2) on November 17, 2021; which revealed the jugular venous system of the left side is not visualized and the right side jugular venous system is prominent and deep veins and dural venous sinuses appear normal which suggestive of congenital agenesis of jugular venous system of left side. Due to financial difficulties, he has not yet undergone an intervention or any other procedure. He still had a persistent headache, but the patient remained constant during the follow‐up visit the following week.

**TABLE 2:** Investigations of the patient

Sl No	Name of the test	Date	Findings	Normal values
01	Creatinine	10.11.2021	2.3 mg/dl	0.74 to 1.35 mg/dl
02	S. Electrolytes			
03	S. Sodium (Na)‐ mmol/L	10.11.2021	141 mmol/L	135–145 mmol/L
04	S. Potassium (k)‐ mmol/L	10.11.2021	3.62 mmol/L	3.5 to 5.0 (mmol/L)
05	Hemoglobin (Hb) (g/dl)	10.11.2021	15.9 gm/dl	14.0–17.5 gm/dl
06	White blood cells (WBC) (/L)	10.11.2021	28.93 × 10^9^/L	4 × 10^9^–11 × 10^9^/L
07	Platelets	21.01.2022	342 × 10^9^	150 to 400 × 10^9^
08	Erythrocyte sedimentation rate (ESR) (mm)	21.01.2022	16 mm/h	1–20 mm/h

## 3 DISCUSSION

Embryological developmental abnormalities cause vascular malformations. Hemangiomas fade away over time. However, vascular abnormalities can develop and last a lifetime. Even though vascular abnormalities are present at birth, they might not have been discovered until adolescence or the elderly.^10^ Venous abnormalities are generally asymptomatic and typically occur in the head and neck region.^11^

Congenital agenesis of the IJV is a very unusual occurrence.^5^ The CT imaging of an instance of IJV agenesis is described in this article. Early detection can help limit damages during medical therapy of neurological conditions, including headaches, and avoid misdiagnosis of other prevalent diagnoses. The IJVs are the prominent venous outflow veins in the brain. In a patient with congenital agenesis of the IJV, impairment of the alternate pathways of vascular supply from the cranial cavity can have catastrophic repercussions.

IJV passes through the neck's carotid sheath and behind the clavicle's sternal end. It then joins the subclavian vein to form the brachiocephalic vein, which travels through the thorax to reach the superior vena cava. Clinically, knowledge of IJV anatomical differences is critical for venous applications.^5^

Different IJV anomalies have been revealed in the scientific literature: partial or total duplication,^12^, ^13^ stenosis, complete occlusion, distortions, and intraluminal structures, such as membranes, webs, and inverted valves.^14^ These are frequently discovered by chance or during neck diagnostic techniques due to a suspicious tumor and are sometimes the consequence of compensatory contralateral IJV enlargement.^5^ Our case was slightly different because he identified IJV agenesis based on the headache evaluation. Our patient had no prior history of any other illnesses or thrombosis history. There was no thrombosis on ultrasonography, CT, or neuroimaging, and the IJV was congenitally agenesis. A quick diagnosis of cerebral arterio‐venous malformations should make it possible to begin treatment before a brain hemorrhage occurs. Headaches linked with cerebral arterio‐venous malformations fall into a particular category that can be identified by particular characteristics.^15^

The major cervical veins and key players in draining cerebral venous blood flow are the two IJVs. Anastomosing venous plexi, which are regarded as the principal collateral pathways that maintain fluent venous drainage when the IJVs are constricted, allow the paired IJVs to physically interact with one another.^16^ Extrajugular venous collaterals will impressively develop to make up for the obstructed principal venous outflow channels in the case of substantial IJV constriction.^16^ In our case, the patient has an absence of left internal jugular vein as it may contribute the brain venous circulation followed by headache.

Patients with anomalies in extracranial venous drainage have shown hemodynamic changes, such as a reduction in cerebral perfusion.^17^, ^18^ There may be a correlation between the degree of brain parenchymal hypoperfusion and the severity of IJV insufficiency.^18^ Moreover, there is proof that improving the aberrant flow caused by immobile jugular leaflets in the IJV can reduce swollen brain ventricles and improve cerebral hypoperfusion. Depletion of glucose and oxygen due to decreased cerebral blood flow (CBF) can result in further negative events such as neuronal mitochondrial malfunction and neural cell loss.^16^ Although it is yet unclear how the IJV anomalies affect brain hemodynamics, it is hypothesized that altered vascular structure and compliance brought on by increased intracranial venous pressure play necessary roles.^19^, ^20^ Strong evidence exists that suggests venous outflow anomalies may play a role in the emergence of intracranial hypertension. Based on individual variance and compensatory capacity, IJV outflow disruption clinical manifestations can range in severity from mild to severe. In general, symptoms including headache, pulsatile tinnitus, vision loss, disturbed sleep, and neck pain or discomfort may at least partially resemble those of chronic cerebral circulation insufficiency and idiopathic intracranial hypertension.^16^, ^21^, ^22^

Since the brain parenchyma lacks nociceptors, pain in nearby tissues such as blood vessels, meninges, muscle fibers, facial structures, and cranial or spinal nerves often causes headaches. The impression of a headache can be caused by stretching, dilatation, constriction, or any nociceptor stimulation within these components.^23^ A thorough evaluation of systems, a description of any former headache disorders, and a detailed report of the current headache should all be obtained during the history. In addition, precise inquiries about any potentially fatal causes of secondary headache should be made since the responses, together with any examination results, will guide any further testing or urgent treatment.^23^ Additionally, studies show that persistently high cerebral venous pressure can cause hyalinosis, stiffness of the venous artery wall, endothelial dysfunction, and disruption of arteriolar control.^24^, ^25^, ^26^

Patients who report to the emergency department frequently complain of headaches. Most of these headaches are harmless, but others have a more organic severe cause. Patients may present with a chronic headache issue that they cannot manage. The history of headache diagnosis is thoroughly addressed, followed by a discussion of headache patients' emergency presentations. Subarachnoid hemorrhage, meningitis, sinusitis, glaucoma, internal carotid artery dissection, and cerebrovascular illness are the causes of worry and a full explanation of the differential diagnosis. Analgesics, NSAIDs, opioids, and end ergotamine formulations are among the drugs discussed for symptomatic headache treatment. However, some individuals with brain AVMs may exhibit symptoms other than bleeding, such as seizures. Headache, also known as localized head pain. Depending primarily on where the AVM is, some individuals may develop progressively severe neurological symptoms, such as severe headaches.^8^ A brain AVM's signs and symptoms can appear at any age, but they often do so between the ages of 10 and 40. Brain tissue may eventually suffer harm from brain AVMs.^8^ In the emergency service, strategies for treating frequent, chronic headaches keep in mind that agenesis of the jugular venous system can play a role—one of the uncommon causes of headaches that we have seen in our instances. Limitations, more advanced intervention can be used to rule out any linkage between internal jugular vein agenesis and headaches, as well as surgical treatment for this result. The following three elements, which together make up the postulated pathophysiology of impaired extracranial venous drainage, are briefly summarized: diminished cerebral blood flow, changed CSF dynamics, and disturbed intracranial microvasculature, among other effects. However, the specific mechanism through which aberrant venous drainage affects cerebral circulation is mostly unknown and has to be further investigated.^16^ The etiology and pathophysiology of IJV outflow disruption are complex and unclear, making treatment difficult. It also raises intriguing issues about whether venous outflow blockage is linked to certain CNS illnesses, such as migraine and headaches, and if so, whether specific treatment plans need to be created for patients with a variety of categories.

It is now considered standard practice to perform a neck dissection on the side that is not affected by the disease to preserve at least one internal jugular vein before compromising the diseased involved vein to minimize the life‐threatening complication of cerebral edema in patients who require bilateral neck dissections or in cases requiring removal of a single internal jugular vein on the side of the disease due to infiltration of the vein.^27^ The symptoms of internal jugular vein stenosis (IJVS), which include headache symptoms, are a collection of generalized signs and symptoms. Internal jugular vein stenosis (IJVS), can be one of IJV agenesis differential diagnoses. As a result, the suppression of external structures (also known as the styloid process) may be one of the most significant causes of IJVS.^28^ Further studies are needed to clarify whether styloidectomy can manage stylo‐jugular Eagle syndrome (ES).^28^

## 4 CONCLUSION

The internal jugular vein is crucial in intensive care units and head and neck surgeries. Handling the internal jugular vein without considering anatomical variances it might be fatal. Early detection of these anomalies allows for necessary measures to avoid patient injury (e.g., discussions with the patient and surgeons about ideal solution anesthetics plans, cannulation sites, monitoring tactics, and thus further inquiry before operation). The most common headache symptom is usually caused by that benign, and it can generally be detected after a comprehensive history and targeted neurologic and overall physical tests. Headache caused by cerebral venous abnormalities is a new issue that all health care workers should be aware. Future research into these unsolved riddles and the exploration of surgical or nonsurgical methods to optimize the treatment for IJV outflow disturbance will give us a profound understanding of this headache and other similar IJV agenesis‐related issues.

## AUTHOR CONTRIBUTIONS

Mohammad Ashraful Amin involved in conceptualization. Mohammad Delwer Hossain Hawlader involved in supervision. Mohammad Ashraful Amin and Sabrina Nahin involved in writing—original draft. Mohammad Ashraful Amin, Sabrina Nahin, and Mohammad Delwer Hossain Hawlader involved in writing—review and editing.

## FUNDING INFORMATION

This research did not receive any specific grant from funding agencies in the public, commercial, or not‐for‐profit sectors.

## CONFLICT OF INTEREST

The authors declare that they have no competing financial interests or personal relationships that could have appeared to influence the work reported in this paper.

## ETHICAL APPROVAL

The article is about a case study. As a result, our Ethics Committee's consent was not required.

## CONSENT

The patient's written informed consent for publishing of this case report, as well as images, was acquired.

## Data Availability

Data can be shared based on the reader's reasonable re‐quest and priority base and some restrictions will apply.
